# A dataset of European banks in performance evaluation under uncertainty

**DOI:** 10.1016/j.dib.2018.11.048

**Published:** 2018-11-14

**Authors:** Simona Alfiero, Alfredo Esposito, Emmanuel Kwasi Mensah, Mehdi Toloo

**Affiliations:** a Department of Management, University of Torino, Italy; b Department of Economics, University of Insubria, Italy; c Department of Systems Engineering, VŠB-Technical University of Ostrava, Czech Republic

## Abstract

The dataset contains financial indicators from the financial statements of 250 banks operating in Europe which are collated for the 2015 accounting year. First, the dataset is split into input and outputs measures. Then the preferred number of inputs and outputs in relation to the total number of data is selected according to the rule of thumb in data envelopment analysis (DEA). The dataset is related to the research article entitled “Robust optimization with nonnegative decision variables: A DEA approach” (Toloo and Mensah, 2018) [1]. The dataset can be used to evaluate the performance of banks and bank efficiency under uncertainty.

## Specifications table

**Table:** 

Subject area	Operations research and management science
More specific subject area	Data envelopment analysis
Type of data	Table, figure
How data was acquired	Obtainable from financial statements of banks from Bureau van Dick – Bankscope database
Data format	Raw, analyzed with descriptive and statistical data
Experimental factors	The sample consists of raw financial data of banks for the accounting year 2016.
Experimental features	Indicators of interest were systematically selected and collated.
Data source location	Global data
Data accessibility	Data is within this article. Also, largely accessible from the database of the current database host, Orbis Bank Focus: https://banks.bvdinfo.com/version-2018810/home.serv?product=OrbisBanks
Related research article	M. Toloo and E. K. Mensah, “Robust optimization with nonnegative decision variables: A DEA approach,” *Comput. Ind. Eng.*, 2018 [1].

## Value of the data

• The raw data contains key financial statements indicators of 250 banks in Europe which were taken from the individual bank׳s financial statement in 2015.
• The data are arranged in order of the largest bank to the smallest bank in terms of assets
• The data is useful for measuring the performance of banks in Europe and for comparative analysis of sub-regional performances and beyond.
• The data can be used by researchers to evaluate a wide range of efficiency measures for the countries under consideration.

## 1 Data

The data comprises financial indicators in the financial statements of 250 public and private banks operating in Europe. Table 1 shows the distribution of these banks according to the sub-region. Including data on indicators such as assets, employees, personnel expenses, equity, loans, net interest income, deposit from banks, operating income and net fees and commission, the detailed financial statements of the banks were obtained from the Bureau van Dick – Bankscope database for the 2015 accounting year. The summary of descriptive statistics of these indicators for each subregion is provided in Table 2, Table 3, Table 4, Table 5. All the financial indicators were measured in millions of Euros with the exception of employees which is measured in actual figures. The total number of employees is defined as the number of banking professionals and the non-banking staff is given employed in the accounting year.

**Table 1:** Classification according to region.

Region	Number of banks	Percentage
Western Europe	129	51.6
Eastern Europe	22	8.8
Northern Europe	33	13.2
Southern Europe	66	26.4
**Total**	**250**	**100**

**Table 2:** Descriptive statistics for Eastern Europe.

Financial indicators	**Mean**	**SD**	**Min**	**Max**
**Inputs**				
Employees	8471.59	7367.41	2952.00	38,203.00
Assets	23,006.93	13,292.19	10,517.04	62,604.63
Equity	2611.49	1672.28	996.40	7097.94
Personnel Expenses	229.99	155.97	92.15	648.82
**Outputs**				
Deposits Banks	1602.50	1216.85	58.00	4484.65
Loans	14,302.75	8981.52	4132.18	43,617.70
Net Income Revenue	619.62	429.78	230.77	1752.56
Operating Income	330.48	239.11	82.44	919.25
Net Fees Commission	239.79	177.35	76.64	676.85

**Table 3:** Descriptive statistics for Northern Europe.

**Financial indicators**	**Mean**	**SD**	**Min**	**Max**
**Inputs**				
Employees	21,295.39	31,900.42	1374.00	129,400.00
Assets	277,515.12	382,760.47	10,231.98	1,526,980.04
Equity	16,442.98	21,190.44	1056.87	89,950.27
Personnel Expenses	1801.82	2941.45	113.09	13,570.41
**Outputs**				
Deposits Banks	27,492.99	41,157.08	208.60	168,902.51
Loans	135,471.03	163,827.56	5097.33	620,171.67
Net Income Revenue	3374.36	4595.32	121.26	18,149.74
Operating Income	2061.49	3509.67	22.89	17,641.53
Net Fees Commission	1197.31	2042.12	29.70	10,785.48

**Table 4:** Descriptive statistics for Southern Europe.

**Financial indicators**	Mean	SD	Min	Max
**Inputs**				
Employees	15,823.02	33,227.16	217.00	193,863.00
Assets	110,047.41	224,459.34	10,267.48	1,340,260.00
Equity	8165.56	16,031.01	226.30	98,753.00
Personnel Expenses	966.23	1944.74	14.20	11,107.00
**Outputs**				
Deposits Banks	17,311.01	34,733.78	81.40	185,459.00
Loans	63,093.07	123,057.73	768.60	758,505.00
Net Income Revenue	1957.18	4806.37	52.30	33,267.00
Operating Income	1176.55	2271.45	20.29	12,628.00
Net Fees Commission	844.13	1796.64	15.40	10,033.00

## 2 Experimental design, materials and methods

Financial statements of banks were first downloaded from the Bankscope database [2]. Then data on the financial indicators mentioned above were compiled from 250 banks financial statements individually and collated. These banks are arranged in descending order of their assets size. Subsequently, for the performance analysis of the banks using the data envelopment analysis (DEA) tool, the financial indicators are split into two samples. The first is the input measures and the second is the output measures.

The separation of the financial indicators into inputs and outputs measures was done based on the selective measures described in [3,4]. The approach adopted for selecting inputs and outputs is the intermediary approach of banking studies, which is shown in Table 2, Table 3, Table 4. With the exception of deposit and loans refereed mostly in literature as dual role factors, it is unarguable the selection of the measures as input and inputs. In this paper, the selection of deposit specifically as output corresponds to its treatment in [4,1]. The number of DMUs in correspondence to the input and outputs measures is selected according to the rule of thumb in DEA as follows (for more details see [5,6]):

n≥max{m×s,3(m+s)}

where

n= total number of DMUs (observations).
m= number of inputs.
s= number of outputs.

All the raw data are scaled for uniformity and to reduce round-off errors from excessively large values prior to analysis.

**Table 5:** Descriptive statistics for Western Europe.

Financial indicators	Mean	SD	Min	Max
Inputs				
Employees	12,144.32	28,624.09	586.00	189,000.00
Assets	141,635.11	339,445.33	10,017.70	1,994,193.00
Equity	7716.21	16,315.15	296.30	100,077.00
Personnel Expenses	961.52	2382.19	64.40	16,061.00
Outputs				
Deposits Banks	20,154.79	45,321.98	331.23	263,121.00
Loans	58,635.75	121,719.18	305.60	735,784.00
Net Income Revenue	1585.57	3628.48	69.20	23,133.00
Operating Income	1179.20	3004.06	34.10	19,805.00
Net Fees Commission	725.82	1711.31	6.60	12,765.00
