# Supplementary material for: A dataset of European banks in performance evaluation under uncertainty
Source: Data Brief. 2018 Nov 14;22:214–7. doi: 10.1016/j.dib.2018.11.048 (PMC6302247; doi:10.1016/j.dib.2018.11.048)
Supplement: Supplementary file 1 — Supplementary material [file mmc1.docx]

The authors declare that they have no conflict of interest and all contributions were duly acknowledged.
